# Splenic contraction and cardiovascular responses are augmented during apnea compared to rebreathing in humans

**DOI:** 10.3389/fphys.2023.1109958

**Published:** 2023-03-07

**Authors:** Gustav Persson, Angelica Lodin-Sundström, Mats H. Linér, Samuel H. A. Andersson, Bodil Sjögreen, Johan P. A. Andersson

**Affiliations:** ^1^ Department of Experimental Medical Science, Lund University, Lund, Sweden; ^2^ Department of Health Sciences, Mid Sweden University, Sundsvall, Sweden; ^3^ Department of Biology, Lund University, Lund, Sweden; ^4^ Department of Clinical Sciences, Malmö, Lund University, Lund, Sweden

**Keywords:** apnea, rebreathing, spleen contraction, hemoglobin concentration, diving response, hypoxia, hypercapnia, oxygen saturation

## Abstract

The spleen contracts during apnea, releasing stored erythrocytes, thereby increasing systemic hemoglobin concentration (Hb). We compared apnea and rebreathing periods, of equal sub-maximal duration (mean 137 s; SD 30), in eighteen subjects to evaluate whether respiratory arrest or hypoxic and hypercapnic chemoreceptor stimulation is the primary elicitor of splenic contraction and cardiovascular responses during apnea. Spleen volume, Hb, cardiovascular variables, arterial (SaO_2_), cerebral (ScO_2_), and deltoid muscle oxygen saturations (SmO_2_) were recorded during the trials and end-tidal partial pressure of oxygen (P_ET_O_2_) and carbon dioxide (P_ET_CO_2_) were measured before and after maneuvers. The spleen volume was smaller after apnea, 213 (89) mL, than after rebreathing, 239 (95) mL, corresponding to relative reductions from control by 20.8 (17.8) % and 11.6 (8.0) %, respectively. The Hb increased 2.4 (2.0) % during apnea, while there was no significant change with rebreathing. The cardiovascular responses, including bradycardia, decrease in cardiac output, and increase in total peripheral resistance, were augmented during apnea compared to during rebreathing. The P_ET_O_2_ was higher, and the P_ET_CO_2_ was lower, after apnea compared to after rebreathing. The ScO_2_ was maintained during maneuvers. The SaO_2_ decreased 3.8 (3.1) % during apnea, and even more, 5.4 (4.4) %, during rebreathing, while the SmO_2_ decreased less during rebreathing, 2.2 (2.8) %, than during apnea, 8.3 (6.2) %. We conclude that respiratory arrest *per se* is an important stimulus for splenic contraction and Hb increase during apnea, as well as an important initiating factor for the apnea-associated cardiovascular responses and their oxygen-conserving effects.

## 1 Introduction

Apnea is a potentially life-threatening consequence of several different involuntary and voluntary situations. Involuntary apnea can occur during accidents such as drowning or choking, but also during clinical conditions such as obstructive sleep apnea, while voluntary apnea is a prerequisite for the sport of breath-hold diving. A transient increase in hemoglobin concentration (Hb) and hematocrit is associated with apnea (Schagatay et al., 2001), which increases the oxygen-carrying capacity of the blood (Stewart and McKenzie, 2002). The increase in Hb and hematocrit during apnea have been explained by a contraction of the spleen, by which it releases its storage of erythrocytes into the circulation (Hurford et al., 1990; Schagatay et al., 2001; Bakovic et al., 2003; Schagatay et al., 2005). The splenic contraction is most likely related to an increase in sympathetic nervous activity, with the splenic nerve containing approximately 98% sympathetic nerve fibers (Felten et al., 1985; Mignini et al., 2003). Splenic contraction can also be initiated by various other stressors associated with an increase in sympathetic activity (Shephard, 2016), such as exercise (Laub et al., 1993; Engan et al., 2014), normobaric hypoxic breathing (Richardson et al., 2008; Pernett et al., 2021), and high-altitude exposure (Schagatay et al., 2020; Holmström et al., 2021). The observations with normobaric hypoxic breathing and high-altitude exposure suggest that hypoxia could be a key stimulus for splenic contraction. However, Lodin-Sundström and Schagatay (2010) showed that during similar levels of hypoxia induced by either apnea or normobaric hypoxic breathing, apnea elicited a greater splenic contraction than normobaric hypoxic breathing, even though the hypoxic exposure had a shorter duration in the case of apnea. This indicates that hypoxia is not the only stimulus for splenic contraction during apnea.

Possible stimuli for spleen contraction during apnea, besides hypoxia, includes the development of hypercapnia (Richardson et al., 2012) and the cessation of respiration in itself, either *via* ceased afferent rhythmic input from thoracic stretch receptors, e.g., pulmonary stretch receptors, or *via* direct signaling from the medullary respiratory center, representing a central sympathetic mechanism which could initiate splenic contraction (Bakovic et al., 2013). The idea that cessation of respiration could be a separate stimulus for initiation of splenic contraction is intriguing, given that rebreathing seems to attenuate the cardiovascular responses induced by apnea (Lin et al., 1983a; Lin et al., 1983b; Lindholm et al., 1999). Rebreathing can be achieved by ventilating in to and out of an air bladder, e.g., an anesthesia balloon, thereby maintaining respiratory movements without gas exchange with the ambient air. Comparable to apnea, rebreathing will be associated with progressive hypoxia and hypercapnia, i.e., reductions in the pulmonary and blood oxygen stores and increases in the pulmonary and blood carbon dioxide stores thus causing hypoxic and hypercapnic chemoreceptor stimulation, but without the cessation of respiratory movements (Lin et al., 1983a). The influence of ceased respiratory movements in stimulating splenic contraction and elevation of Hb can thus be examined by comparing periods of apnea and periods of rebreathing. To what extent the cessation of respiration in itself is of importance for splenic contraction has not been addressed in any previous study.

In addition to initiating splenic contraction, apnea initiates a characteristic pattern of cardiac and vascular responses, which are the outcome of several reflex mechanisms occurring simultaneously with interactions between these reflexes (Gooden, 1994). Collectively, this pattern of responses is called the diving response. The human diving response is characterized by bradycardia, reduced cardiac output (CO), peripheral vasoconstriction and increased arterial blood pressure, and is elicited by apnea with or without concomitant facial chilling (Foster and Sheel, 2005). While there is a preponderance of data showing that apnea, alone or combined with face immersion, initiates the cardiovascular changes associated with the diving response, there is only a limited amount of data concerning the potential effects that rebreathing have on modifying the cardiovascular responses to apnea in resting humans, especially when rebreathing is initiated using ambient air as the rebreathing gas. Lin et al. (1983a, 1983b) reported that rebreathing attenuated the heart rate and circulatory responses initiated by apnea with face immersion. However, to what extent there is a difference in the responses between rebreathing and apnea alone (without face immersion) was not reported. Lindholm et al. (1999) reported that the heart rate and blood pressure responses to apnea were attenuated by rebreathing in experiments involving steady-state exercise.

The cardiovascular responses of the diving response affect the pulmonary gas exchange and distribution of blood oxygen stores. Reduced CO during apnea reduces the oxygen uptake from the lungs (Linér and Linnarsson, 1994; Lindholm and Linnarsson, 2002; Andersson et al., 2004; Andersson et al., 2008). Reduced peripheral blood flow together with increased blood flow in the carotid arteries and increased velocity in the middle cerebral artery during apnea reflect a redistribution of blood flow towards the brain (Pan et al., 1997; Leuenberger et al., 2001; Palada et al., 2007). Also, muscle oxygen desaturation occurs earlier than arterial oxygen desaturation, and cerebral oxygenation is preserved longer than muscular oxygenation during apnea (Valic et al., 2006; Bouten et al., 2020), supporting the view of an appropriate blood flow redistribution. Taken together, this supports the hypothesis of oxygen-conserving effects of the diving response, by supplying vital organs with oxygen while peripheral oxygen stores are depleted (Andersson et al., 2004). To what extent rebreathing affects the oxygen-conserving effects of the cardiovascular responses in resting humans is unknown.

In order to investigate the stimuli for and initiation of spleen contraction and the cardiovascular diving response in humans, we measured spleen volume and Hb, as well as recorded cardiovascular responses, during repeated periods of apnea and repeated periods of rebreathing. We hypothesized that the reduction in spleen volume and increase in Hb would both be greater during periods of apnea if ceased respiratory movements is an important stimulus for splenic contraction. We also hypothesized that the cardiovascular diving response would be attenuated during rebreathing compared to apnea and that the oxygen-conserving effects of the responses would be reduced if cessation of respiratory movements is an important initiating factor for the cardiovascular diving response.

## 2 Materials and methods

### 2.1 Ethics approval statement

The protocol was reviewed and approved by the Swedish Ethical Review Authority (2019-05435) and all trials were conducted in conformity with the principles of the Declaration of Helsinki. With the recruitment of volunteers, they were provided written information about, e.g., the procedures, potential risks, and handling of data. At the laboratory, after verbal clarification of test procedures and potential risks involved, the participants provided their oral and written informed consent to participate in this study. Exclusion criteria were age below 18 or above 45 years, any acute or chronic disease, use of medications (with the exception of contraceptives), and pregnancy. Anomalies in blood pressure or on 12-lead electrocardiogram (ECG), controlled before the start of each trial, would lead to discontinuation of the trial.

### 2.2 Subjects

Eighteen healthy volunteers (15 male, 3 female) were recruited for the study. None of the subjects were smokers, but two were users of snuff (smokeless tobacco). The subjects’ mean (SD, range) age was 28 years (7, 21–43), height 180 cm (8, 164–191), weight 79 kg (13, 58–103), and forced vital capacity 5.6 L (0.9, 3.9–7.3). All subjects had previous experience of breath-hold diving or apnea, and 13 subjects were active breath-hold divers or underwater-rugby players, training at least 2 h/week. Their physical training, besides diving activities, averaged 5 h/week (4, 1–15). For active breath-hold divers or underwater-rugby players, their self-reported maximal breath-holding time was 236 s (77, 60–340), and years of breath-holding experience was 8 years (8, 1–22). All subjects arrived at the laboratory after at least 2 h without any heavy meal or caffeine-containing beverages, with only light physical activity being performed within 12 h of the trial.

### 2.3 Protocol

Upon arrival at the laboratory [air temperature 22.2°C (0.9), ambient pressure 758.9 mmHg (7.4), relative humidity 26.7% (5.3)], thorough verbal information about the procedures were given and the equipment that was to be used was demonstrated. All participants were given the opportunity to ask any questions about the procedures before signing the informed consent form for participation in the study, as well as completing a health questionnaire. Height and weight of the subject were initially measured. Blood pressure was measured in the seated position to ensure normal blood pressure before the start of the trial. Forced spirometry was performed with the subject in the standing position. The subject then assumed a supine position on a mattress, and a 12-lead ECG was recorded and assessed for anomalies. With cushioned support, the subject was placed in a slightly slanted position so that the left, thoracoabdominal, dorsolateral area was exposed to allow sonographic imaging of the spleen. This supine position was maintained for the remainder of the experimental protocol. Again, the vital capacity was measured, now with the subject in the described supine position. Following this, a peripheral venous catheter, used for venous blood sampling, was inserted in an antecubital vein. Thereafter, non-invasive probes for measuring continuous cardiovascular data, arterial oxygen saturation (SaO_2_), cerebral oxygen saturation (ScO_2_), and deltoid muscle oxygen saturation (SmO_2_) were attached. The subject rested in the supine position for a minimum of 20 min before any tests began, to ensure stabilization of the transcapillary fluid exchange and blood mixing (Lundvall and Bjerkhoel, 1994), thus ensuring a stable, baseline Hb.

**FIGURE 1:**
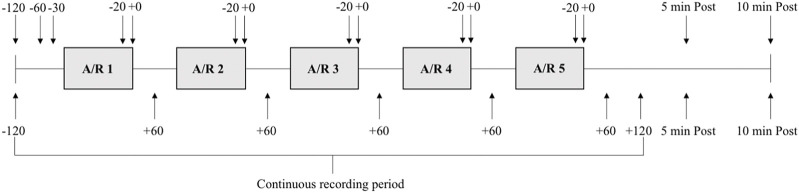
Schematic overview of the experimental protocol. Each grey box indicates a period of apnea or rebreathing (A/R). Each subject performed one series of five apneas and one series of five rebreathing episodes. Solid lines between boxes indicates 2 minutes of rest between each maneuver. Arrows with time stamps (seconds) indicate when ultrasound measurements (above boxes) and venous blood samples (below boxes) were collected in relation to each apnea or rebreathing, as well as five (5 min Post) and 10 min (10 min Post) after termination of the fifth apnea or rebreathing in the series.

In a crossover study design, each subject performed two series of tests, i.e., one series of five apneas and one series of five rebreathing periods (A vs. R, Figure 1). Half of the subjects started with the series of apneas and half with the series of rebreathing periods. The series were separated by 20 min of rest. Within series, tests were spaced by 2-min intervals. The duration of all tests was predetermined to be the same for each individual subject. The individual subjects’ test duration was agreed upon following discussion with the subject, based on maximal apneic attempts performed at home prior to participating in the trial (three separate sessions, with three repeated apneas within each session). The duration was set so that the subject felt comfortable being able to complete the first period of apnea while the remaining apneas still would be strenuous. Most individuals are expected to be able to perform rebreathing for longer durations than apnea, as performing a rebreathing maneuver, even without improving the alveolar gas composition, reduces the respiratory distress during a breath-hold (Flume et al., 1994). I.e., apnea, and not rebreathing, was considered to be the limiting type of test from a subjective perspective. Also, the duration was set in regard to the first period of apnea as there is a short-term training effect of repeated apneas (Heath and Irwin, 1968; Schagatay et al., 1999). The subject was instructed to avoid hyperventilation before tests, and to stay relaxed before, during, and after the tests.

Each series was initiated by a 2-min recording period, and recordings continued throughout the whole series. Spleen volume measurement and blood sampling for determination of Hb were collected at specified time points during the series (see below). At 30 s before each test a nose-clip was attached, and a mouthpiece from an open-circuit spirometry system was inserted into the subject’s mouth, to enable collection of end-tidal gases. At 10 s before tests, the subject exhaled to the residual volume through the mouthpiece and was then immediately switched to a mouthpiece connected to a pre-filled air bladder, and inhaled 85% of the supine vital capacity, marking the start of the test. During apneas, the mouthpiece connected to the air bladder was removed, and the subject held the inhaled volume of air in the lungs until the breaking point of apnea. During rebreathing, the mouthpiece was kept in the mouth, and the subject respired freely into and from the air bladder, trying to maintain eupneic ventilation. Time cues were given by the experimenter during the tests according to prior consultation with the subject, and a countdown was performed by the experimenter during the last 10 s of the tests. Ten seconds prior to termination of apneas, i.e., with the countdown, the mouthpiece of the open-circuit spirometry system was inserted into the subject’s mouth, while still holding the breath. Ten seconds prior to termination of rebreathing all the remaining air in the air bladder was inspired and then the subject was immediately switched from the air bladder mouthpiece to the mouthpiece of the open-circuit spirometry system, while holding the breath. At the end of the countdown, upon command from the experimenter, apneas and rebreathing was terminated by the subject expiring to residual volume through the mouthpiece of the open-circuit spirometry system. The mouthpiece and the nose-clip were removed, and the subject rested, remaining in the supine position between tests.

### 2.4 Measurements and data collection

A wall-mounted height measurer and an electric scale (BF214, Omron Healthcare Europe, Hoofddorp, Netherlands) were used to measure height and weight, respectively. Pre-trial blood pressure in the seated, resting position, was measured using an automatic sphygmomanometer (Boso-medicus, Bosch + Sohn GMBH, Jungingen, Germany). A hand-held spirometer (Micro plus, Micro Medical Ltd, Rochester, England) was used for spirometry measurements in both the standing and supine positions. To avoid any risks associated with the technique, glossopharyngeal insufflation was not allowed during spirometric testing or the rest of the protocol (Andersson et al., 2009a; Linér and Andersson, 2010). Twelve-lead ECG was recorded using an ECG-monitor (Cardiovit AT-1 G2, Schiller, Doral, FL, United States).

Ultrasound measurements of the spleen were performed on 13 of the 18 subjects, using an ultrasonic device (Sonosite M-Turbo, FUJIFILM Sonosite Inc, Bothell, WA, United States), with the transducer C60x/5-2 MHz (Transducers, Sonosite Inc, Bothell, WA, United States). Ultrasound measurements of the spleen were performed by an experienced sonographer (AL-S) at 120 s, 60 s, and 30 s before the initiation of the first test in each series, 20 s before termination of each test, at termination of each test, and additionally five and 10 min after termination of the fifth test of each series (Figure 1). Two images were taken for every time point to determine maximal length (*L*), maximal thickness (*T*) and maximal width (*W*) of the spleen. Spleen volume was calculated from the collected measurements of *L*, *T* and *W* using the equation developed by Pilström (Schagatay et al., 2005): *L*π(*WT*-*T* ^2^)/3.

Venous blood for measurements of Hb was collected at 120 s prior to the start of each series, 60 s after termination of each test, and additionally five and 10 min after termination of the fifth test of each series (Figure 1). Blood samples were collected 60 s after termination of tests because any potential elevation in Hb due to splenic contraction was expected to lag, as venous blood was collected peripherally, and to avoid any influence from peripheral vasoconstriction during apneas inhibiting successful blood sample collection (Andersson et al., 2004). The catheter was flushed with a small volume of isotonic saline solution after each blood sample. Approximately 15 mL blood was drawn from each subject, including “waste samples” that were collected just before each measurement sample to avoid any residual saline solution in the catheter influencing the Hb measurements. Venous blood Hb was photometrically determined with three microcuvettes for each blood sample (Hb 201 + Analyzer, HemoCue AB, Ängelholm, Sweden), using the average value from the triplicates.

Continuous recordings of cardiovascular parameters and O_2_ saturations began 2 minutes prior to the first test and continued until 2 minutes after the end of the test (Figure 1). Heart rate (HR), stroke volume (SV), cardiac output (CO), total peripheral resistance (TPR), and arterial blood pressures were recorded continuously using a finger photoplethysmograph (Finapres NOVA, Finapres Medical Systems BV, Enschede, Netherlands). The Finapres NOVA monitoring system measures beat-by-beat finger arterial pressure using a finger cuff with a built-in photoplethysmograph (Bogert and van Lieshout, 2005). The finger arterial pressure is calibrated using the Physiocal algorithm, and the finger pressure is reconstructed into brachial arterial pressure, applying waveform filtering and level correction, which in turn is calibrated with a brachial blood pressure cuff. The Finapres NOVA uses the Modelflow^®^ algorithm to calculate cardiovascular parameters, such as SV, CO, and TPR from the recorded finger arterial pressure (Wesseling et al., 1993; Bogert and van Lieshout, 2005). The reconstructed arterial brachial pressure was recorded during the trials. The finger cuff and the brachial cuff were placed on the middle finger and over the brachial artery, respectively, on the same upper extremity during the trials. One subject was excluded from subsequent analysis of HR due to problems with data acquisition.

The SaO_2_ was continuously measured using a finger pulse oximeter (Biox 3700e, Ohmeda, Madison, WI, United States), with the probe placed on the index finger. The ScO_2_ was recorded, every 4 seconds, using a regional oximeter (Nonin SenSmart Model X-100 Universal Oximetry System, Nonin Medical, Plymouth, MN, United States), with an adhesive probe (SenSmart Equanox 8204CA rSO_2_ sensor, Nonin Medical, Plymouth, MN, United States) attached on the left side of the forehead, lateral of the superior sagittal sinus, superior to the eyebrow, and inferior to the hairline. Likewise, the SmO_2_ of the deltoid muscle was recorded using the same regional oximeter and same type of probe, using another channel, with the probe attached 5 cm below the acromion. One subject was excluded from subsequent analysis of SmO_2_ due to problems with data acquisition.

Ambient air temperature, barometric pressure, and relative humidity in the laboratory, as well as end-tidal O_2_ and CO_2_ concentrations, were measured using an open-circuit spirometry system (Ergocard Professional, Medisoft, Sorinnes, Belgium). End-tidal partial pressure of O_2_ (P_ET_O_2_) and end-tidal partial pressure of CO_2_ (P_ET_CO_2_) were calculated from the measurements of end-tidal O_2_ and CO_2_ concentrations from the expirations to residual volume, just before and at the end of each test. The open-circuit spirometry system was calibrated using a 3-L syringe (Hans Rudolph, Shawnee, KS, United States) and certified gases (AGA Gas, Lidingö, Sweden) prior to the start of each trial.

The above cardiovascular and respiratory variables were all recorded onto personal computers using a data acquisition system (MP100, BIOPAC Systems, Inc., Goleta, CA, United States) and stored for later analysis using AcqKnowledge, Excel, and SPSS.

### 2.5 Data analysis

The control spleen volume was defined as the mean of spleen volumes 120 s, 60 s, and 30 s before initiating the first test in each series. Apneic values and rebreathing values of spleen volume were defined as the mean of spleen volumes measured 20 s before termination of each apnea or rebreathing and at termination of each apnea or rebreathing. For one of the subjects, measurements 20 s before termination of apnea or rebreathing were not collected, and for one other subject, two of these measurements were not collected. In these two subjects, apneic values and rebreathing values of spleen volume were based on the available measurements. Data are presented in absolute values and as relative changes from control during apnea and rebreathing, respectively.

The control Hb was defined as the value of the blood collected 120 s before initiating the first test in each series. Apneic values and rebreathing values of Hb were defined as the mean value of the blood collected 60 s after termination of each apnea and rebreathing. Data are presented in absolute values and as relative changes from control during apnea and rebreathing, respectively.

Control values for HR, SV, CO, TPR, and blood pressures were calculated as mean values from the period 60–30 s prior to each test. Apneic values and rebreathing values for these variables were calculated as mean values from the period 20–10 s before the end of each test. This period was chosen because the cardiovascular changes elicited by apnea has been reported to be more stable during the later parts of apnea (Jung and Stolle, 1981; Perini et al., 2008). The last 10 s of apnea and rebreathing were excluded because of the fitting of the mouthpiece of the open-circuit spirometry system during this period. Results are presented as group mean absolute values and group mean relative changes from control.

Comparable to the cardiovascular variables, control values for SaO_2_, ScO_2_, and SmO_2_ were calculated as mean values during the period 60–30 s prior to each test. The nadir of SaO_2_ during the 40-s period immediately after each test was identified. Apneic values and rebreathing values for ScO_2_ and SmO_2_ were calculated as mean values from the period 20–10 s before the end of each test. Results are presented as group mean absolute values and group mean relative changes from control.

The P_ET_O_2_ and P_ET_CO_2_ of the final expiration to residual volume prior to each test were calculated as one apneic pre-value and one rebreathing pre-value for each subject. Likewise, the P_ET_O_2_ and P_ET_CO_2_ of the expiration to residual volume ending each test were calculated as one apneic post-value and one rebreathing post-value for each subject. Results are presented as group mean absolute values.

For each subject, individual mean values from the five apneas and five rebreathing periods, respectively, were calculated for all variables, and group means of the individual means were calculated. IBM SPSS Statistics for Windows, Version 28.0 (IBM Corp, Armonk, NY) was used to perform statistical analysis. Data was checked for normal distribution, using Shapiro-Wilk test, before statistical tests were performed. For data following a normal distribution, paired sample, two-tailed *t*-test was used to compare apnea and rebreathing (between-series comparisons), and one-way repeated measures analysis of variance with Bonferroni corrected *t*-test was used to compare spleen volume and Hb at different time points during the apnea and rebreathing series, respectively (within-series comparisons). For data not following a normal distribution, Related-Samples Wilcoxon Signed Rank Test was used to do between-series comparisons and Friedman test with Bonferroni corrected Wilcoxon Signed Rank Test was used to do within-series comparisons. Values reported in the text are means (SD). The level used for accepting significance was *p* < 0.05.

## 3 Results

### 3.1 Duration of apnea and rebreathing periods

The duration of apneas and rebreathing periods were identical within each subject, with a mean duration of 137 (30) seconds, ranging from 90 s to 200 s. All subjects managed to complete each apnea and rebreathing period to the predetermined time.

### 3.2 Spleen volume and hemoglobin concentration

**FIGURE 2:**
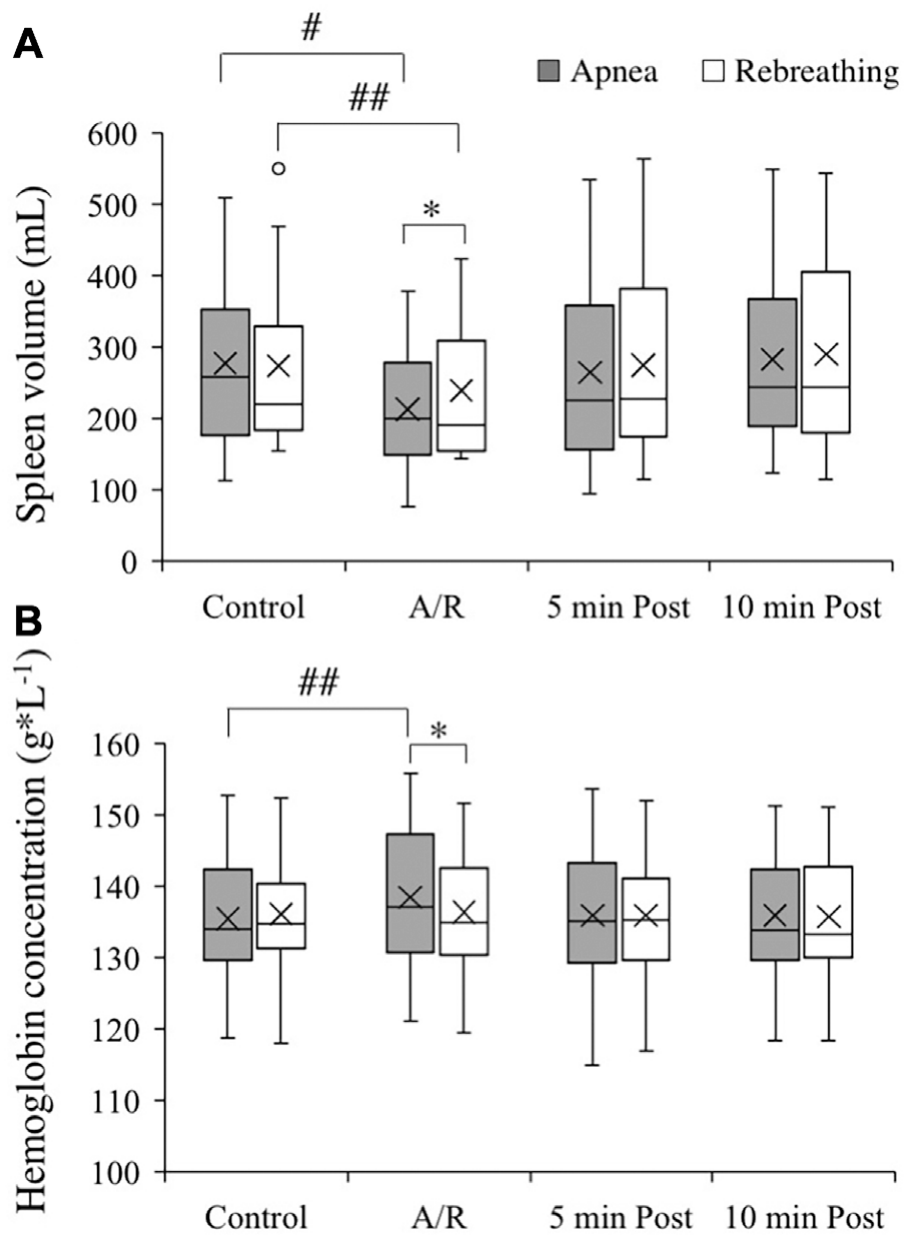
Spleen volume [**(A)**, *n* = 13] and hemoglobin concentration of venous blood [**(B)**, *n* = 18] before apneas or rebreathing (Control), during the series of apneas or rebreathing (A/R), and five and 10 min after termination of the fifth apnea or rebreathing (5 min Post and 10 min Post, respectively). The boxes indicate the first and third quartile of the data with the median (vertical line) and mean (X) values indicated. The whiskers indicate the lowest and the highest data points in the data set, excluding any outliers (circles). *p < 0.05 between apnea and rebreathing. #*p* < 0.05 and ##*p* < 0.01 compared with respective control value.

The control spleen volume before the apnea series and rebreathing series did not differ, 277 (125) and 274 (121) mL, respectively (Figure 2, *p* = 0.92 A vs. R). The spleen volume decreased from control both during the series of apnea and series of rebreathing periods, 213 (89) and 239 (95) mL, respectively (*p* < 0.05 vs. control), and returned to the control level within 5 minutes after the last apnea and rebreathing period in their respective series (*p* = 1.00 vs. control). The spleen volume remained at the control level 10 min after the series (*p* = 1.00 vs. control). Comparing apnea and rebreathing periods regarding the absolute values for spleen volume during the tests, the spleen volume was smaller after periods of apnea (*p* = 0.04 A vs. R). Expressed as relative changes, the mean spleen volume decreased 20.8 (17.8) % from control during apnea and 11.6 (8.0) % during rebreathing (*p* = 0.14 A vs. R).

The control Hb for the apneic and the rebreathing series did not differ, 135 (9) and 136 (8) g/L, respectively (Figure 2, *p* = 0.60 A vs. R). The Hb increased from control during the series of apneas, 139 (9) g/L (*p* < 0.01 vs. control), while it did not change significantly from control throughout the rebreathing series, 137 (8) (*p* = 0.38 vs. control). Five minutes after the end of the apneic series, the Hb had returned to the control level and remained there at 10 min after the end of the series (both *p* = 1.00 vs. control). Comparing apnea and rebreathing periods regarding the absolute values for Hb during the tests, the Hb was higher after periods of apnea (*p* = 0.03 A vs. R). Expressed as relative changes, the mean Hb increased 2.4 (2.0) % from control during the series of apnea, which was a greater change than during the rebreathing series (0.4 (1.4) %; *p* < 0.01 A vs. R).

### 3.3 Cardiovascular responses

**FIGURE 3:**
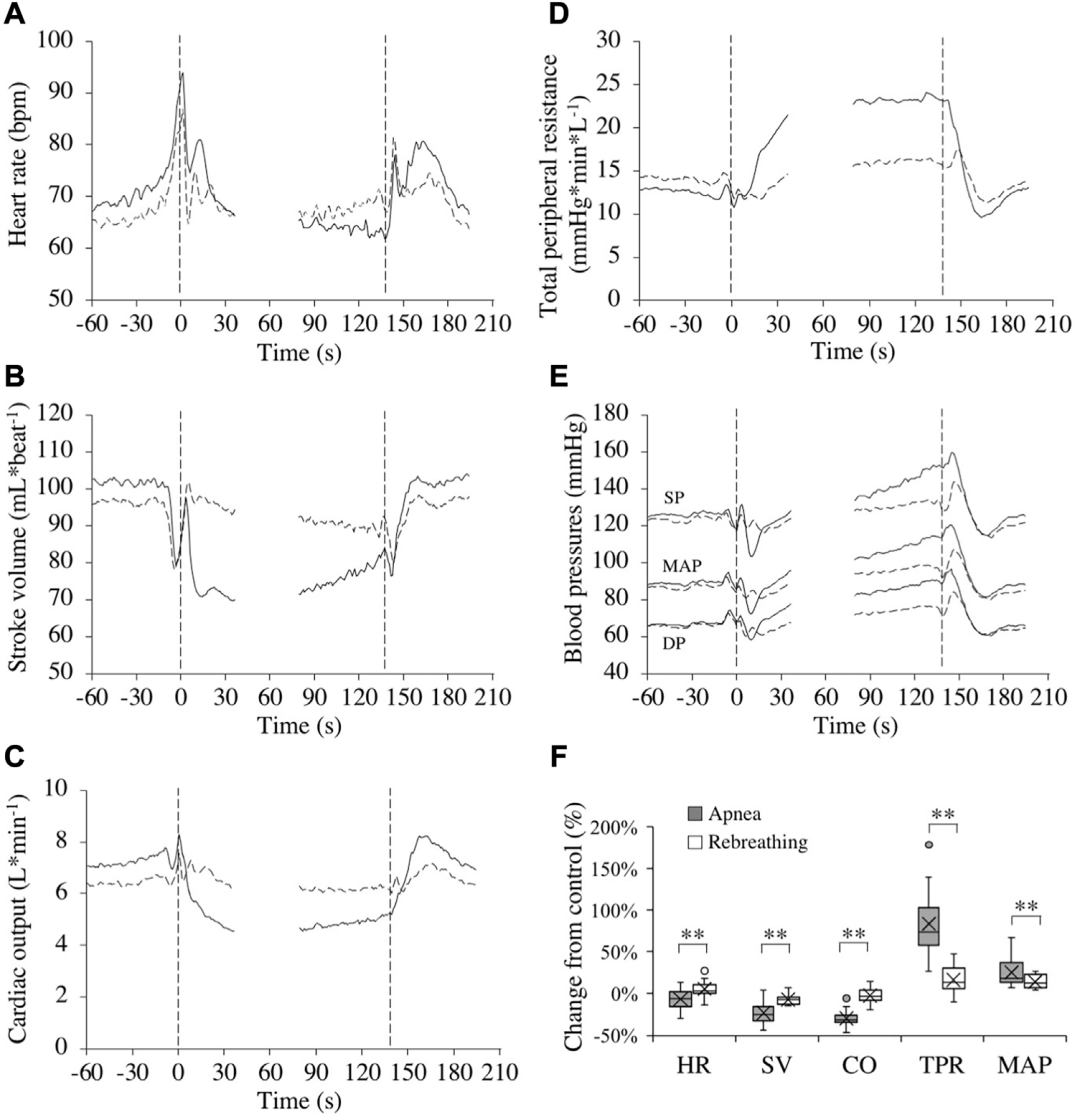
Cardiovascular responses (*n* = 17 or 18, see text) in association with apneas (solid lines) and rebreathing periods (dashed lines). Panel 3 **(A–E)** show the means of each variable from before tests (–60–0 s), during the first 35 s of tests (0–35 s), during the last 60 s of tests (78–137 s), and the first 60 s after tests (138–198 s). Vertical dashed lines indicate the start and end of apneas and rebreathing periods. Breaks in the lines reflect the fact that test durations varied among the subjects, and the position of the end of tests in the graphs has been adjusted for each subject to match the average duration of the tests (137 s). Error bars have been omitted for clarity. Panel 3 **(F)** shows boxplots for the relative changes in each variable (HR, heart rate; SV, stroke volume; CO, cardiac output; TPR, total peripheral resistance; MAP, mean arterial pressure) during 20–10 s before the end of apneas and rebreathing periods compared to their respective control value (see figure legend 2 for details). **p < 0.01 between apnea and rebreathing.

Control values for the cardiovascular variables before apneas and rebreathing periods, respectively, are presented in Table 1. For HR, after reaching a peak value right at the onset of tests (Figure 3A), there was a bradycardic response with apnea, which was attenuated by rebreathing. Towards the end of the tests, the HR had decreased from control during apnea by −6.4 (11.0) % (*p* = 0.03 vs. control) and increased from control during rebreathing by +5.4 (9.3) % (*p* = 0.04 vs. control), with a significant difference between apnea and rebreathing (*p* < 0.01 A vs. R, Figure 3F). Similarly, the SV and CO were both reduced by apnea (Figures 3B,C), with a quick reduction in SV with the initiation of apnea. At 20–10 s before the end of apneas, the reductions from control in SV and CO were −23.3 (11.6) and −29.6 (9.0) %, respectively (both *p* < 0.001 vs. control, Figure 3F). With rebreathing, the SV was gradually reduced, albeit at a much slower rate than during apnea (Figure 3B). Towards the end of the tests, the SV had decreased from control during rebreathing by −7.1 (5.7) % (*p* < 0.001 vs. control), while there was no significant change in CO with rebreathing, −2.6 (8.9) % (*p* = 0.18 vs. control, Figure 3F). I.e., the changes in SV and CO were attenuated by rebreathing so that at 20–10 s before the end of tests the reductions from control in these variables were larger during apnea than during rebreathing (*p* < 0.001 A vs. R, Figure 3F). Within the first 30 s of apnea, there was a noticeable increase in TPR, which was maintained for the duration of apnea, while the increase in TPR was attenuated during rebreathing (Figure 3D). As with the other cardiovascular responses, the change in TPR from control with apnea, +83.9 (36.4) %, was larger than the change with rebreathing, +16.4 (15.3) % (both *p* < 0.001 vs. control, *p* < 0.001 A vs. R, Figure 3F). The blood pressure gradually increased throughout the tests, but more evidently during apnea than during rebreathing (Figure 3E). The increase from control in MAP was larger during apnea, +25.4 (15.7) %, than during rebreathing, +13.8 (7.6) % (both *p* < 0.001 vs. control, *p* < 0.001 A vs. R, Figure 3F).

**TABLE 1:** Control values for cardiovascular variables and oxygen saturations, recorded 60–30 s before the onset of each apnea or rebreathing period.

	Apnea	Rebreathing	
HR (bpm)	68.4 (8.7)	64.8 (8.4)	*p* < 0.01
SV (mL*beat^-1^)	102.0 (25.1)	96.6 (22.4)	*p* = 0.01
CO (L*min^-1^)	7.1 (2.0)	6.4 (1.7)	*p* < 0.01
TPR (mmHg*min*L^-1^)	12.9 (5.5)	14.2 (5.6)	*p* < 0.01
MAP (mmHg)	88.3 (12.7)	86.0 (12.2)	*p* = 0.13
SaO_2_ (%)	98.5 (1.1)	98.5 (1.1)	*p* = 0.68
ScO_2_ (%)	73.0 (6.9)	73.5 (6.4)	*p* = 0.35
SmO_2_ (%)	81.5 (6.5)	79.7 (6.9)	*p* < 0.01

Values are means (SD), n = 18 (HR, and SmO_2_, n = 17). HR, heart rate; SV, stroke volume; CO, cardiac output; TPR, total peripheral resistance; MAP, mean arterial blood pressure; SaO_2_, arterial oxygen saturation; ScO_2_, cerebral oxygen saturation; SmO_2_, deltoid muscle oxygen saturation.

### 3.4 End-tidal oxygen and carbon dioxide

The P_ET_O_2_ and the P_ET_CO_2_ before apneas and rebreathing, respectively, did not differ (*p* = 0.06 A vs. R and *p* = 0.09 A vs. R, respectively, Table 2). The P_ET_O_2_ decreased and the P_ET_CO_2_ increased during the tests. The P_ET_O_2_ was lower after periods of rebreathing than after periods of apnea of identical durations (*p* < 0.001 A vs. R). Likewise, the P_ET_CO_2_ was higher after periods of rebreathing than after periods of apnea (*p* < 0.001 A vs. R).

**TABLE 2:** End-tidal partial pressure of oxygen and carbon dioxide before and after apnea or rebreathing.

	Apnea	Rebreathing	
Pre-P_ET_O_2_ (mmHg)	118.7 (7.0)	117.1 (7.1)	*p =* 0.06
Post-P_ET_O_2_ (mmHg)	71.3 (9.9)	59.8 (9.6)	*p* < 0.001
Pre-P_ET_CO_2_ (mmHg)	32.8 (4.3)	33.5 (4.3)	*p =* 0.09
Post-P_ET_CO_2_ (mmHg)	45.2 (3.6)	47.4 (3.9)	*p* < 0.001

Values are means (SD), n = 18. Pre-P_ET_O_2_ and Pre-P_ET_CO_2_ were collected during the final expiration before each apnea and rebreathing. Post-P_ET_O_2_ and Post-P_ET_CO_2_ were collected during the expiration terminating each apnea and rebreathing. P_ET_O_2_, End-tidal partial pressure of oxygen; P_ET_CO_2_, End-tidal partial pressure of carbon dioxide.

### 3.5 Arterial, cerebral, and muscle oxygen saturations

**FIGURE 4:**
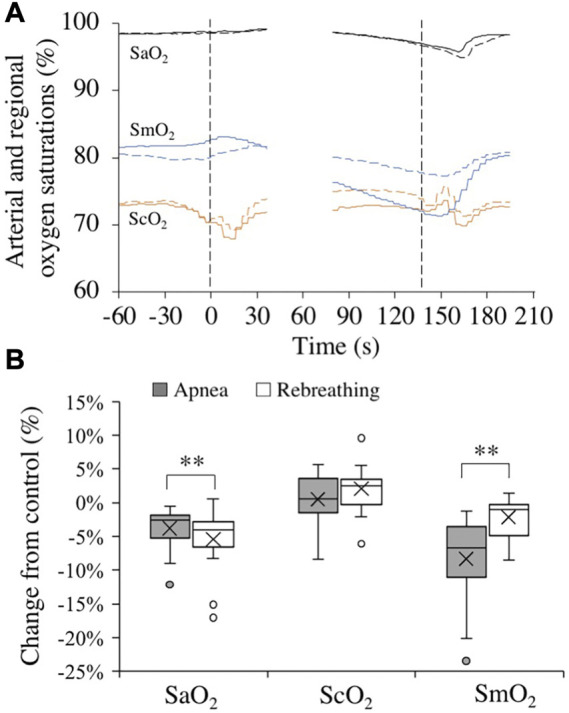
Changes in arterial (black, SaO_2_, *n* = 18), cerebral (orange, ScO_2_, *n* = 18), and deltoid muscle (blue, SmO_2_, *n* = 17) oxygen saturations in association with apneas (solid lines) and rebreathing periods (dashed lines). Panel 4 **(A)** shows the means of each variable from before, during, and after tests (see figure legend 3 for details). Panel 4 **(B)** shows boxplots for the relative changes in each variable at nadir (SaO_2_) or during 20–10 s before the end of apneas and rebreathing periods (ScO_2_ and SmO_2_) compared to their respective control value (see figure legend 2 for details). **p < 0.01 between apnea and rebreathing.

Control values for arterial and regional O_2_ saturations before apneas and rebreathing periods, respectively, are presented in Table 1. Towards the end of the tests, the SaO_2_ was reduced (Figure 4A). At nadir in SaO_2_, occurring after the end of tests, there was a larger decrease from control with rebreathing, −5.4 (4.4) %, compared to with apnea, −3.8 (3.1) % (both *p* < 0.001 vs. control, *p* < 0.01 A vs. R, Figure 4B). The ScO_2_ was maintained during apnea and increased from control during rebreathing (Figure 4A). Towards the end of the tests, the ScO_2_ had increased from control during rebreathing by +2.0 (3.4) % (*p* = 0.03 vs. control), while there was no significant change in ScO_2_ with apnea, +0.5 (3.5) % (*p* = 0.58 vs. control), with no significant difference between apnea and rebreathing (*p* = 0.10 A vs. R, Figure 4B). Within the first 30 s of apnea, the SmO_2_ began to fall, while during rebreathing there was no reduction in SmO_2_ during the initial 30 s of the test (Figure 4A). At the period 20–10 s before the end of tests, the reduction in SmO_2_ was greater during apnea, −8.3 (6.2) %, compared to during rebreathing, −2.2 (2.8) % (both *p* < 0.01 vs. control, *p* < 0.001 A vs. R, Figure 4B).

## 4 Discussion

There are three main conclusions that are supported by the findings of the present study. First, apnea elicited a greater contraction of the spleen and a greater increase in Hb than rebreathing, even though the chemoreceptor stimuli from hypoxia and hypercapnia were augmented by rebreathing, indicating that apnea is an important stimulus for the splenic and hematological responses. Second, apnea was associated with augmented cardiovascular responses in comparison to rebreathing. Lastly, the cardiovascular responses initiated by apnea were associated with oxygen-conserving effects, indicated by the observations that the pulmonary and arterial oxygen levels were preserved to a greater extent, while at the same time SmO_2_ was reduced more, during apnea compared to during rebreathing. There are no previous studies in which these variables have all been studied in resting humans using the protocol of the present study, with apnea and continuous rebreathing of the same prolonged duration, starting with normoxic and normocapnic gas in the air bladder. As such, the present study adds knowledge to the understanding of human splenic, hematologic, and cardiorespiratory responses to apnea, and how these are initiated.

### 4.1 Splenic contraction and hemoglobin concentration

Expressed in absolute values, the spleen volume decreased from control during both apnea and rebreathing, and the spleen volume was smaller during the series of apneas than during the series of rebreathing periods. As control spleen volume did not differ before the two maneuvers and spleen volume was smaller during apnea compared to rebreathing, apnea elicited a more powerful contraction of the spleen than rebreathing. Expressed in relative values, even though the spleen volume decreased by almost 10 percentage points more during apnea than during rebreathing, the difference in relative change from control between the two conditions did not reach statistical significance in this sample. This was potentially caused by low statistical power in the analysis of spleen volume, as the spleen measurements were limited to 13 subjects and the standard deviation was relatively large. With the decrease in spleen volume, the Hb increased from control during periods of apnea. However, during rebreathing, when the spleen volume was larger than during apnea, the Hb did not increase. Taken together, the results support the notion that with a more pronounced splenic contraction, there is a greater release of stored erythrocytes. In addition, the results support the hypothesis that the absence of continued respiratory movements in itself is an important stimulus for initiating splenic contraction during apnea.

Previous studies have found effects from chemoreceptor stimulation on splenic contraction and associated hematological changes (Pernett et al., 2021). Lodin-Sundström and Schagatay (2010) showed that apnea induced a significantly more pronounced splenic contraction than normobaric hypoxic breathing, at similar hypoxic stress, and suggested that hypercapnia or a faster development of hypoxia during apnea might explain the difference. Richardson et al. (2009) showed that hypoxia augments the increase in hematocrit and Hb during apnea. In contrast, the results of the present study show that even with more pronounced hypoxia and hypercapnia during rebreathing, as evident by the end-tidal gases, the splenic contraction was augmented during apnea, and rebreathing failed to increase the Hb. As the duration of both maneuvers were the same, hypoxia likely developed more rapidly during rebreathing compared to apnea in the present study. Thus, if anything, the finding of augmented hypoxia and hypercapnia with rebreathing underestimates the importance of the respiratory arrest *per se* for initiating the splenic and hematological changes during apnea. The augmented splenic and hematological responses elicited by apnea could be related to either ceased cyclic afferent input from pulmonary stretch receptors, from direct signaling from the respiratory center or a higher brain center, or by a combination of these mechanisms. Nevertheless, chemoreceptor input could contribute to modulating splenic contraction during apnea as there was a splenic contraction also with rebreathing in the present study. Additionally, the contribution from chemoreceptor input is indicated by the observations that hematocrit and Hb increases more during hypoxic apneas than during normoxic apneas (Richardson et al., 2009), and that Hb tend to increase more during hypercapnic apneas than during hypocapnic apneas (Richardson et al., 2012). But in the face of continued respiratory movements, the chemoreceptor input seems to be less important compared to the act of apnea for actually initiating splenic contraction and expulsion of erythrocytes. Thus, it seems as if apnea is a more potent initiator of sympathetic nervous activity to the spleen than the chemoreceptor stimulation provided by rebreathing. This conclusion is in line with the observations that sympathetic responses, such as increases in TPR and MAP, are greater during apnea than during rebreathing, both in the present study and the studies by Badrov et al. (2017) and Marullo et al. (2022). Also, Steinback et al. (2010) found that apnea induced peripheral vasoconstriction and a pressor response, with a muscle sympathetic nerve activity response, which were all attenuated during rebreathing, further lending support to the notion that apnea could be more effective in triggering sympathetically-induced splenic contraction than rebreathing.

The observed decrease in spleen volume of approximately 21% during apnea in the present study was in agreement with earlier studies utilizing repeated periods of apnea under similar experimental conditions (e.g., Bakovic et al., 2003; Schagatay et al., 2005; Prommer et al., 2007; Engan et al., 2013), but smaller than the decrease observed in elite breath-hold divers, 48% (Schagatay et al., 2012). The increase in Hb in the present study, 2.4% during apnea, is also similar to what have been reported in previous studies (e.g., Richardson et al., 2005; Schagatay et al., 2005; Bouten et al., 2019). However, there is a large variation in reported values regarding how the Hb is acutely affected by apneas, from no change (Prommer et al., 2007; Dolscheid-Pommerich et al., 2020) to an increase by 8% (Andersson et al., 2009b). This variation can most likely be attributed to differences in type of subjects included, experimental conditions, apneic protocols, e.g., number of apneas, duration of apneas and intervals between apneas, as well as when in relation to the end of apneas that blood samples were collected. During recovery from the series of apneas in the present study, the spleen volume and Hb had returned to control within 5 minutes, which are similar but slightly faster time courses than what previously have been observed by Schagatay et al. (2005). They reported complete restoration of spleen volume after 9 minutes of recovery from three maximal-duration apneas, whereas in the present study repeated submaximal apneas were used. It is possible that a series of maximal-duration apneas elicit a more durable contraction of the spleen than submaximal apneas. It was recently reported that, following contraction induced by a single maximum-duration apnea in subjects inexperienced in apneic diving, spleen volume had reached the baseline, pre-apnea volume after 5 minutes (Holmström et al., 2022). Also, Schagatay et al. (2005) reported that Hb had returned to control after 10 minutes of recovery. However, they did not report measurements of Hb in between one and 10 minutes of recovery. It is thus possible that Hb had returned to control earlier than after 10 minutes of recovery. The fast recovery of spleen volume and Hb is in agreement with previous observations that the short-term training effect of repeated apneas is lost after 10 minutes, and that elevated hematocrit and Hb, *via* splenic contraction, is at least partly causative for the short-term training effect (Schagatay et al., 1999; Schagatay et al., 2001).

### 4.2 Cardiovascular changes

The cardiovascular diving response was attenuated during rebreathing periods compared to apneas. This was observed for all variables recorded in the present study. Even though the apneic and rebreathing control values for HR, SV, CO, and TPR differed (Table 1), the differences were small and unlikely to explain much of the observed differences between apnea and rebreathing.

In the present study, HR decreased slightly during periods of apnea and increased slightly during periods of rebreathing, in line with earlier observations (Badrov et al., 2017). However, these findings are somewhat in contrast to observations by Lin et al. (1983b), reporting a decrease in HR during rebreathing and a substantially larger apneic bradycardia compared to the one observed in the present study. The larger apneic bradycardia in the previous study can most likely be explained by the augmented HR-response induced by face immersion (Lin et al., 1983b), as the protocol used by Lin et al. (1983b) included apneas with face immersion while the apneas in the present study were performed with the face in air. Apneic bradycardia of the same magnitude as in the present study have been observed in previous studies with apneic protocols similar to this study (e.g., Andersson and Schagatay, 1998). With regard to the difference in HR response to rebreathing between the study by Lin et al. (1983b) and the present study, it should be noted that in addition to the face immersion, the rebreathing protocol of the previous study involved a set 15-s interval between breaths, whereas the rebreathing protocol in the present study was conducted with “free”, untimed ventilation. A 15-s interval between breaths during rebreathing is probably long enough to partially initiate a bradycardic response between breaths during rebreathing, since the HR decrease begins within seconds of beginning a period of apnea (Jung and Stolle, 1981; Perini et al., 2008). Thus, this potentially explains why the HR on average increased during rebreathing in the present study and decreased in the study by Lin et al. (1983b).

The SV was reduced during both apnea and rebreathing, but the change was both much faster and augmented during apnea in the present study. This is in contrast with some earlier studies on apnea and rebreathing (Lin et al., 1983a; Badrov et al., 2017). Lin et al. (1983a) observed an increase in SV during both apnea and rebreathing, while Badrov et al. (2017) observed no significant changes in SV, neither during apnea, nor during rebreathing. As with HR, explanations for the differing observations can most likely be explained by differences in the experimental protocols. In the study by Badrov et al. (2017), apneas were performed at functional residual capacity, whereas in the present study, apneas were performed at a rather large lung volume (85% of supine VC). As intrathoracic pressure increases when apnea is performed with a larger lung volume and relaxed respiratory muscles, SV is expected to decrease with increased held lung volume during apnea, following an impediment of venous return (Ferrigno et al., 1986). Thus, the different observations regarding SV in the present study and the study by Badrov et al. (2017) can be explained by the differences in lung volumes at the beginning of apneas. The lung volume at which apneas were performed in Lin et al. (1983a) is not specified, and therefore the precise reason for our different observations cannot be determined. However, in earlier studies of apneas performed at a lung volume above the functional residual capacity recurrent observations of reductions in SV have been reported (Ferrigno et al., 1986, 1987; Costalat et al., 2013; Fagoni et al., 2015; Magnani et al., 2018), which is further supported by the observation of reduced SV during apnea in the present study, a response which seems to be largely negated by rebreathing. With the continued ventilation during rebreathing, there is no continuous elevation in intrathoracic pressure, and thus the venous return should not have been impeded during rebreathing to the same extent as during apnea, explaining the observed differences in time course for, and magnitude of, the SV reduction between apnea and rebreathing in the present study.

With the observed changes in HR and SV, the CO was markedly reduced from control during apnea, in accordance with earlier studies including recordings of CO during apnea in resting humans (Ferrigno et al., 1986; Costalat et al., 2013). However, during rebreathing there was no significant change in CO, showing that the continued respiratory movements attenuate the cardiac responses to apnea, even in the face of chemoreceptor activation. As discussed below, this difference in CO between apnea and rebreathing is probably a major factor explaining the difference in the pulmonary oxygen stores at the end of the two tests.

The TPR increased both during apnea and rebreathing periods, but substantially more during apnea, which is in agreement with previous findings (Badrov et al., 2017). Badrov et al. (2017) showed that continued respiratory movements inhibit the apnea-associated sympathetic activation, even at high levels of hypoxia and hypercapnia. Augmented elevation of TPR during apnea compared to during rebreathing in the present study, even though chemoreceptor activation was more pronounced during rebreathing, support the observations of Badrov et al. (2017). Thus, the sympathetic responses to apnea seems to be attenuated by continued respiratory movements. The increase in TPR is related to a peripheral vasoconstriction, leading to a redistribution of blood flow towards the brain (Pan et al., 1997; Leuenberger et al., 2001; Palada et al., 2007). The results of the present study support that this redistribution of blood flow is augmented during apnea compared to during rebreathing.

With the changes in CO and TPR, the MAP was increased from control during apnea as well as during rebreathing. The MAP did however increase significantly more during apnea than during rebreathing, an expected effect of the attenuated rise in TPR during rebreathing. Similar findings have previously been reported (Badrov et al., 2017; Marullo et al., 2022), and this further support that continued respiratory movements attenuate the sympathetic responses to apnea. It is likely that the increase in cardiac afterload, by the observed increases in arterial pressure in the present study, contributed to the reduction in SV during the apneas and rebreathing periods.

#### 4.3 End-tidal oxygen and carbon dioxide, arterial and regional saturations

Rebreathing periods induced lower P_ET_O_2_ and higher P_ET_CO_2_ compared to apneas with identical durations in the present study. The influence of chemoreceptor activation on splenic contraction and the diving response was thus probably greater during rebreathing periods than during apnea. The observed smaller splenic volume and augmented cardiovascular diving response during apnea compared to rebreathing must therefore have been an effect of the apnea *per se* rather than an effect of the chemical stimuli of hypoxia and hypercapnia.

In the healthy lungs, the pulmonary gas exchange is perfusion limited. Thus, changes in the pulmonary oxygen and carbon dioxide stores during apnea and rebreathing are to a great extent dependent on changes in the CO. Following this line of reasoning, the higher P_ET_O_2_ after apnea compared to rebreathing in the present study could be explained by the decrease in CO during apnea, as a decrease in CO with a simultaneous redistribution of systemic blood flow and peripheralization of venous blood volume is accompanied by a decrease in oxygen uptake from the lungs (Linér and Linnarsson, 1994). With an attenuated reduction in CO, as during rebreathing, the pulmonary oxygen store is depleted at a faster pace. Another factor that could have influenced the difference in P_ET_O_2_ after the maneuvers was the oxygen cost of the respiratory work being performed during rebreathing periods, but not during apneas. For the sake of discussion, pulmonary oxygen uptake ( $\dot{V}$ O_2_) was estimated by determining the volume of gases in the air bladder and the lungs (residual volume) at the start and end of apnea and rebreathing, respectively. The subject’s residual volume was obtained from a normal values formula (Grimby and Soderholm, 1963). This calculation showed that the $\dot{V}$ O_2_ during apnea was 87% of the $\dot{V}$ O_2_ during rebreathing. As the oxygen cost of breathing normally is less than 5% of the total $\dot{V}$ O_2_ (Kanak et al., 1985), the lower P_ET_O_2_ after rebreathing can not only be explained by the oxygen cost of breathing. This support the conclusion that the cardiovascular responses during apnea contributed to reducing the oxygen uptake from the lungs, thereby having an oxygen-conserving effect that was attenuated during rebreathing.

In the present study, SaO_2_ and SmO_2_ decreased during apnea, which is in accordance with earlier observations (Andersson and Schagatay, 1998; Valic et al., 2006; Bouten et al., 2020). The SmO_2_ fell more than SaO_2_ during apnea, likely due to the marked rise in TPR, restricting the blood flow to peripheral tissues, in this case the deltoid muscle, and thus causing a greater tissue, venous blood, desaturation, an observation that has previously been made by Valic et al. (2006). The SaO_2_ and SmO_2_ also decreased during rebreathing in the present study, but the SaO_2_ decreased significantly more than during apnea while the SmO_2_ decreased less. The greater decrease in SaO_2_ with rebreathing compared to with apnea is in agreement with the greater decrease in P_ET_O_2_ with rebreathing, which is, as explained above, likely attributable to the difference in CO. As the increase in TPR was attenuated during rebreathing compared to during apnea, the peripheral blood flow and the peripheral oxygen delivery remained relatively higher during rebreathing compared to during apnea, explaining the higher SmO_2_ during rebreathing. Combined, these observation shows that the cardiovascular diving response has an oxygen-conserving effect and that this effect was attenuated during rebreathing, which is in agreement with what Lindholm et al. (1999) showed during exercise. The ScO_2_ remained unchanged from control during apnea, which is in contrast with previous studies (Palada et al., 2007; Bouten et al., 2020), who have reported a fall in ScO_2_ from control. These previous studies used protocols with maximum apneas and had average apnea durations of 157 s and 244 s (breath-hold divers), respectively (Palada et al., 2007; Bouten et al., 2020), while the present study used submaximal apneas with an average apnea duration of 137 s. Thus, the submaximal apneas of the present study were probably not long enough to induce a fall in ScO_2_. Towards the end of the rebreathing periods the ScO_2_ had increased from control. This could potentially be an effect of the more pronounced hypercapnia during rebreathing, evident by the higher P_ET_CO_2_, as hypercapnia causes an increase in cerebral blood flow (Ito et al., 2003).

Taken together, the smaller reductions in P_ET_O_2_ and SaO_2_ during apnea compared to during rebreathing, combined with maintained ScO_2_ and markedly reduced SmO_2_ during apneas, reflect the oxygen-conserving effect of the cardiovascular diving response, with reduced oxygen uptake from the lungs, reduced peripheral blood flow, and increased cerebral blood flow (Linér and Linnarsson, 1994; Pan et al., 1997; Leuenberger et al., 2001).

#### 4.4 Methodological considerations

Spleen volume was estimated from ultrasonic measurements using an equation (Pilström) that has not been directly validated against other measurement methods, such as magnetic resonance imaging or direct measurements of resected spleens. However, the Pilström equation has been validated against other equations used for ultrasonic determinations of spleen size, which in turn have been validated using spleen volume measurements conducted *via* autopsy (Lodin-Sundström, 2015; Holmström et al., 2022). Also, the Pilström equation has been reported to display high test–retest reliability (Holmström et al., 2022), especially when using mean values from repeated measurements (several time points), as in the present study. Furthermore, the Pilström equation has been used to estimate spleen volume in several other studies investigating splenic contraction during apnea (Schagatay et al., 2005; Lodin-Sundström and Schagatay, 2010; Richardson et al., 2012; Lodin-Sundström, 2015; Elia et al., 2021), and was therefore chosen in order to make comparisons with previous studies possible. Considerations regarding the Pilström equation has been extensively reviewed by Lodin-Sundström (2015).

Finger photoplethysmography is an indirect method for measuring cardiovascular variables, which makes it susceptible to errors (Wesseling et al., 1993; Bogert and van Lieshout, 2005). There are other methods available, such as magnetic resonance imaging or invasive catheterization that would produce more accurate measurements of cardiovascular variables. However, as the data extracted from the finger photoplethysmograph was primarily used for relative comparisons within subjects, the accuracy of the measurements was not as important as potential errors are likely to be similar within the same subject. Also, a stress-free environment was desirable during the trials, to avoid any influence of the experimental setting on the results. Finger photoplethysmography is a non-invasive method which only uses a finger cuff and a brachial blood pressure cuff and was therefore likely to induce minimal discomfort or stress for the subjects. With the mentioned limitations, it should be noted that the cardiovascular observations in the present study are comparable to observations reported in other studies, using similar or alternative methods for measuring cardiovascular variables (e.g., Perini et al., 2010; Breskovic et al., 2011; Costalat et al., 2013; Sivieri et al., 2015).

Regional oximetry was used to evaluate cerebral and muscle oxygen saturations. As opposed to values obtained using pulse oximetry, corresponding to the arterial oxygen saturation, regional oximetry reflects both the arterial and venous oxygen saturations (Steppan and Hogue, 2014), with an arterial to venous blood contribution to the signal of 30% and 70%, respectively. As such, the regional oximetry signal is influenced by factors such as the oxygen content of arterial blood, regional blood flow, and regional oxygen consumption, thus providing information on the balance between regional oxygen supply and demand (Steppan and Hogue, 2014). We do not expect the cerebral and deltoid muscle oxygen demand to change during the maneuvers in the present study. Thus, reported changes in regional oximetry are assumed to most likely reflect variations in the regional oxygen supply, i.e., the oxygen content of the arterial blood and the regional blood flow. With the simultaneous recording and evaluation of the arterial oxygen saturation, changes in cerebral and muscle oxygen saturations in the present study allowed evaluations of changes in cerebral and deltoid muscle blood flows, even though these were not directly measured. From a qualitative perspective, our observations and conclusions are in line with previously reported changes (Pan et al., 1997; Valic et al., 2006; Palada et al., 2007; Bouten et al., 2020).

## 5 Conclusion

This study shows that apnea is an important stimulus for splenic contraction, resulting in elevation of the Hb, and that cessation of respiratory movements rather than chemoreceptor activation during apnea is the major trigger for splenic contraction and Hb increase. This study also shows that apnea, *via* cessation of respiratory movements, is an important initiating factor for several of the components of the cardiovascular diving response and that the cardiovascular changes are associated with oxygen-conserving effects during apnea. The results from this study can however not conclude whether it is ceased cyclic stimuli from, e.g., pulmonary stretch receptors, direct signaling from the respiratory center or a higher brain center, or a combination of these that are important during apnea to initiate splenic contraction and the cardiovascular diving response.

## Data Availability

The raw data supporting the conclusion of this article will be made available by the authors, without undue reservation.
